# A Wonderful Peruvian Railroad

**Published:** 1885-11

**Authors:** 


### A Wonderful Peruvian Railroad.

One of the most wonderful pieces of engineering in the world is the railroad stretching from Lima and Callao to the crest of the continent, where the famous mines of the Cerro del Pasco are the source of the ancient riches of the country, from which tons upon tons of silver have been taken and which still hold, if the testimony of the mineralogist can be relied upon, the richest deposits on the surface of the world. The railroad was never completed. Mr. Meigs carried it from Lima to the crest of the Andes at a cost of $27,000,000 and 7,000 human lives, and gained for himself a reputation for energy and ability surpassing any man that ever came to this continent, but he died with about fifty miles of track yet to be laid.

No one has been found with the courage to finish the work until a few weeks ago Michael Grace, of New York, whose brother and partner in the enterprise is the mayor of that city, made a contract with the government, under the terms that he is to be given the road as it stands, with all its equipments, if he will complete it to its original destination. He agrees to complete the remaining fifty miles of railroad and pump out of the mines of Cerro del Pasco the water that has been accumulating in them for half a lazy century, in consideration for which the government gives him that portion of the road already completed, and all the silver he can get out of the mines during the next ninety-nine years, he paying the nominal rental of $25,000 a year for the use of the property.

The sensation of riding up this railroad, together with the rapid ascent from the sea level to the mountain's crest, produces a sickness called "sirocche," often fatal, and usually sending people to bed for several weeks. The symptoms are a terrible pressure upon the temples, nausea, bleeding of the nose and ears and faintness, but the effect can be avoided by taking precautions and observing rules that experience has suggested, the chief one being to drink a glass of brandy and keep perfectly quiet, as the slightest degree of exercise will floor the strongest man. People who are compelled to make the ascent, if they have not become accustomed to it, usually take two or three days f r the journey, stopping off at the stations along the line, and going to bed at once upon reaching the town of Chicla, which stands at the summit.—*Philadelphia Times.*

---

ACCORDING to the *American Naturalist* we are to have a new kind of potato that will not rot. It is to be a hybrid formed between the common Irish potato and a similar tuber found in the southern-most parts of South America. Next to a roll of fresh dairy butter that has never flirted with the oleaginous portions of the festive porker the potato that has no decay lurking in its system will occupy the front door of the American household.

---

THE RUMFORD CHEMICAL WORKS, Providence, R. I., manufacturers of Prof. Horsford's Acid Phosphate, have recently purchased a commodious building and warehouse near their present location, where they propose to move their business a few months hence. This purchase has been necessitated by the demands of their large and increasing business, and it is pleasant to record such an evidence of well deserved success and prosperity.

